# Comparison of inpatient distribution amongst different medical alliances in a county: a longitudinal study on a healthcare reform in rural China

**DOI:** 10.1186/s12939-020-01260-x

**Published:** 2020-08-20

**Authors:** Yifan Ran, Hongxia Gao, Dan Han, Guilin Hou, Yingchun Chen, Yan Zhang

**Affiliations:** 1 School of Medicine and Health Management, Tongji Medical College, Huazhong University of Science and Technology, Wuhan, 430030 Hubei China; 2 Research Centre for Rural Health Service, Key Research Institute of Humanities and Social Sciences of Hubei Provincial Department of Education, Wuhan, 430030 Hubei China

**Keywords:** Medical alliances (MAs), Inpatient distribution, Interrupted time series analysis (ITSA), Rural health, Health inequality

## Abstract

**Background:**

China has launched the medical alliances (MAs) reform to drive the development of primary medical institutions and decrease health inequality in rural areas. Three different types of MAs were built to promote township hospitals in Y County. This study aims to evaluate the actual effect of China’s MAs reform in rural areas on inpatient distribution especially amongst different types of MAs.

**Methods:**

We obtain 2008–2015 claims data from the New Cooperative Medical Scheme (NCMS) in Y County, Hubei Province of China. We consider January 2008–December 2010 as the pre-reform period and January 2011–December 2015 as the post-reform period.

We use independent sample t-test and single-group interrupted time series analysis (ITSA) to compare the number of inpatients per month in the three MAs, including three county and 10 township hospitals before and after the reform. We use paired t-test and multiple-group ITSA between seven township hospitals within MAs and seven township hospitals outside MAs.

**Results:**

The MAs reform in Y County increased the number of inpatients in county and township hospitals within MAs. After the reform, the number of inpatients per month in county hospitals had an upward trend, with a slope of 31.01 person/month (*P* < 0.000). Approximately 19.99 new inpatients were admitted to township hospitals monthly after the reform (*P* < 0.000). Furthermore, township hospitals within MAs had a substantial increase in the number of inpatients (10.45 new inpatients monthly) compared with those outside MAs.

**Conclusion:**

The MAs reform in Y County significantly improved the capability of medical institutions within MAs. After the reform, township hospitals within MAs had greater development advantages than those outside MAs. However, it also caused further imbalance in the county region, which contained the new health inequality risk.

## Background

How to improve the efficiency of limited health resources is a common problem worldwide, especially in the current situation where the aging society and high incidence of chronic diseases entail a surge in medical demand. As a country with large population, China, including the urban and rural regions, is facing great difficulties in healthcare.

Guiding patients to seek medical treatment in an orderly manner and reducing the number of medical visits to large hospitals can effectively reduce expenditures on health services [1]. China’s county health system has adopted a three-level network to provide effective health service since the 1950s, namely, county-, township- and village-levels. And the county-level hospitals were divided into three types, namely, People’s Hospital (PH), Chinese Medicine Hospital (CMH) and Maternal and Child Health Hospital (MCH). Relatively clear functional orientation existed amongst different county-level hospitals and between county and township hospitals when the three-level health service network system was designed and in the initial stage of practice [2]. Taking the inpatient service as an example, the township hospital was responsible for common disease treatments, whereas the county hospital diagnosed complex diseases. Generally, the medical expenses of county-level hospitals were significantly higher than those of primary medical institutions. However, regardless of the severity of the disease, rural residents were more willing to move to county hospitals to seek medical advice due to the reality of ability limitations of primary health institutions [3]. In addition, farmers have always been a vulnerable group in China in terms of health. Therefore, the three-level health service network did not function well. Inefficient use of health resources and significant health inequality still existed within the county region. In 2009, China launched the new medical reform and simultaneously begun to build the primary healthcare system to improve the capability of primary medical institutions. Unfortunately, the allocation of medical resources between large and primary hospitals remained unbalanced [4, 5].

Ultimately, the Chinese government drew from the international experience of Integrated Delivery Networks (IDN) as well as domestic pilot practices and established Medical Alliances (MAs) with full-featured, well-defined and resource-sharing projects [6]. The policy was aimed at driving the development of primary medical institutions through large public hospitals to achieve coordinated development and ultimately minimise the health inequality throughout the region. MAs refer to the integration of medical information and resource sharing by integrating vertical and horizontal resources and form different medical health collaboration alliances or medical groups in a certain area [7]. This model continuously improves the overall efficiency in medical service organisations and is vital for primary medical institutions to develop capabilities and its collaborative service functions [8].

China’s MAs reform is different from its international counterpart, such as the IDN in the USA. IDN contains vertical combinations, including acquisition of primary care physicians (PCPs), strategic alliances with physicians in physician–hospital organizations (PHOs) and management services organizations (MSOs), the development of health maintenance organizations (HMOs) and horizontal combinations, including the formation of multihospital systems, mergers and strategic alliances with neighbouring hospitals to form local networks [9]. However, MAs in China are a direct vertical merger between county and township hospitals. Besides, the goal of IDN is to pursue higher economic benefits by achieving scale effect, whereas the primary goal of MAs in China is to decrease health inequality by improving the capability of primary medical institutions. Given that public hospitals occupy a dominant position in China’s healthcare system, the government plays a comprehensive guiding role in the MAs reform [10]. Therefore, government’s mandatory characteristics are evident in China’s MAs reforms.

Many provinces and cities in China have explored various MAs modes in urban [11, 12] and in rural areas [13, 14]. In the reform practice of rural MAs, three kinds of county hospitals have always been the leading hospitals, and the specific reform can be divided into two categories. The first involves merging all county hospitals into one large hospital, such as in F County, Anhui Province [15]. The other is to build three MAs, with each county hospital being responsible for several township hospitals as a leading hospital, such as Y city in Hubei province.

At present, we cannot directly judge which of the two approaches of MAs suits rural China better, but we can attempt to analyze some potential influence of the operation of MAs on the medical resources from the perspective of the three-level rural health network system. According to the MAs policy, three different types of county hospitals were responsible for several township hospitals and formed three communities of interests. This reform seemed like a way to stimulate the capacity of leading hospitals and township hospitals. However, the capacity of three county hospitals were unbalanced, and PH was often the strongest hospital. We are concerned that the MAs reform will widen the gap between the three county hospitals and even cause greater competency gaps amongst the three MAs due to different capabilities of leading hospitals. Meanwhile, hospitals within MAs were not closely allied in terms of benefits, personnel or equipment use. Although hospitals within MAs were all public hospitals, their financial support from the government was very limited and each hospital needed economic income to support its development. Therefore, competition amongst hospitals within MAs cannot be avoided. We are also concerned that whether the leading hospitals will seize resources from township hospitals within MAs even after the reform due to interest issues.

Our study aims to analyze the effect of the MAs reform on inpatient service distribution, especially amongst different types of MAs in county region. We selected Y County as the sample area where MAs reform has been carried out as a pilot. In 2010, Y County launched a MAs reform wherein three MAs were built on the basis of three county hospitals, and each county hospital managed several township hospitals. Medical Alliance one (MA1) consisted of PH and four township hospitals. Medical Alliance two (MA2) consisted of CMH and three township hospitals. Medical Alliance three (MA3) consisted of MCH and three township hospitals [16]. The reform was completed at the end of 2010. After the reform, 14 township hospitals were outside MAs. We used the number of inpatients as the main indicator to evaluate the actual effect of MAs reform.

## Methods

### Study design and data sources

The study design was based on a retrospective comparative study. We collected the data from the NCMS database of 322,521 inpatient medical records in Y County, which were completed by 27 hospitals from 2008 to 2015, including three county and 24 township hospitals. In the MAs reform in 2010, three county and 10 township hospitals formed three MAs and the other 14 township hospitals were not included in the reform.

This design required comparing the number of inpatients in three county and 10 township hospitals within MAs before and after the reform and further comparing the number of inpatients in seven township hospitals within MAs and seven outside MAs after the reform. We compared seven township hospitals within MAs with seven township hospitals outside MAs, because there were seven special towns where there was one township hospital within MAs and one township hospital outside MAs. Therefore, instead of comparing 10 township hospitals within MAs with 14 township hospitals outside MAs, we compared these seven selected township hospitals within MAs as an intervention group and seven selected township hospitals outside MAs as a non-random comparison group. We took this method to design these two groups, mainly because the different development trends of the two types of township hospitals before and after the reform can better reflect the actual effect of the reform, especially in the same town.

Y County began to implement NCMS in 2007. At the end of 2015, the registered population of Y County was 562,577 and the participation rate of NCMS was 100% [17]. NCMS only had claims for those rural residents in the county and not for those residents who lived in the county but had urban ID registration. Urban residents obtained their health insurance through urban resident or employee medical insurances [18].

We collected the inpatient database of NCMS in Y County from January 1, 2008–December 31, 2015. The database included demographic information (i.e. patient’s age, gender, nationality, residence, residence code and whether or not he is the head of the household), hospitalisation information (i.e. hospitalisation and discharge time, days of hospitalisation, disease name and code, total hospitalisation expenses, reimbursable expenses and actual reimbursement expenses), NCMS ID, hospital coding and other information.

This study focuses on the hospitalisation situation of country farmers. Thus, we selected and retained the hospitalisation data in the county and deleted out-of-county hospitalisation data. The missing items of the demographic and hospitalisation information were deduced and filled logically. We obtained all eligible members’ data and collected a total of 322,521 valid information.

### Variables and outcomes

The core variable is the number of inpatients per month. The MAs reform began in June 2010 and was completed by the end of 2010. Considering the time needed for the reform to advance, this study selected January 2011 as the time boundary for the reform as the policy indicator variable (0, period before the reform is January 2008–December 2010; 1, period after the reform is January 2011–December 2015).

We counted the number and proportion of inpatients from three MAs and three county hospitals from 2008 to 2015. And then we calculated the level and trend of the number of inpatients in three county and 10 township hospitals within MAs before and after the reform.

Furthermore, as we mentioned above, two types of township hospitals existed in one town, one of which was included in the MAs reform and the other one was not. And there were seven such towns in Y County. To further explore the effect of reform on township hospitals, we took the seven township hospitals within MAs as an intervention group and the other seven township hospitals as a non-random comparison group. We mainly measured the two groups by comparing the slope of the number of inpatients per month after the reform.

### Independent sample and paired t tests

Using SPSS 23.0 software, we used independent sample t-test to compare the number of inpatients per month before and after the reform of the three county and 10 township hospitals within MAs. We used paired t-test to compare the number of inpatients in the intervention and non-random comparison groups before and after the reform. *P* < 0.05 indicates statistically significant difference. We used a single sample K–S test to test for normal distribution.

### Interrupted time series analysis (ITSA) for single and multiple groups

Using Stata 14 software, we included the number of inpatients per month in three county and 10 township hospitals from 2008 to 2015 in an ITSA for single-group. The core variable was the number of inpatients per month.

1 $$ {Y}_{\mathrm{t}}={\beta}_0+{\beta}_1{T}_{\mathrm{t}}+{\beta}_2{X}_{\mathrm{t}}+{\beta}_3{X}_t{T}_t+{\in}_t $$

*Y*_t_ denotes the number of inpatients in month t; *T*_t_ is a continuous variable indicating the number of months from the beginning of observation period to time t; *X*_t_ is a variable about t before the reform (*X*_t_ = 0) and after the reform (*X*_t_ = 1) with the 37th month as the demarcation point of reform time. In this model, *β*_0_ estimates the baseline level of core variable at t = 0; *β*_1_ estimates the baseline trend of core variable before the reform; *β*_2_ estimates the immediate change of core variable after the reform; and *β*_3_ estimates the trend change of core variable after the reform. *β*_1_ + *β*_3_ indicates the slope of core variable after the reform. Through this model, the baseline level and trend can be effectively controlled, and then, the level and trend changes caused by the reform can be analysed. The error term *ϵ*_*t*_ at time t represents the random variability not explained by the model. It consists of a normally distributed random error and an error term at time t that may be correlated to errors at preceding or subsequent time points [19, 20].

The number of inpatients per month in the intervention and non-random comparison groups from 2008 to 2015 was incorporated into ITSA for multiple groups. The principle of the model is the same. *Z* is a pseudo-variable representing the non-random comparison and the intervention groups (Z = 0 in the non-random comparison group and Z = 1 in the intervention group).

2 $$ {Y}_{\mathrm{t}}={\beta}_0+{\beta}_1{T}_{\mathrm{t}}+{\beta}_2{X}_{\mathrm{t}}+{\beta}_3{X}_t{T}_t+{\beta}_4Z+{\beta}_5{ZT}_t+{\beta}_6{ZX}_t+{\beta}_7{ZX}_{\mathrm{t}}{T}_t+{\in}_t $$

Given that seasonal difference would cause considerable information to be lost and the trend map of time series showed that seasonal characteristics were not significant, we did not carry out a seasonal adjustment. We conducted the regression with Newey–West standard errors for autocorrelation. The comparability of the core variable in the two groups in terms of the baseline level and baseline trend must be guaranteed to ensure the accuracy of the ITSA model for the multiple groups. Comparability was defined as *β*_2_ and *β*_3_, that both satisfy *P* > 0.10 [20]. Therefore, seven towns must be screened on the basis of comparability.

## Results

### Distribution of inpatients in MAs and county hospitals in Y County

We calculated the distribution of inpatients of 27 medical institutions in Y County from 2008 to 2015, including three county hospitals, 10 township hospitals within MAs and 14 township hospitals outside MAs, hoping to make an overall reflection on the service capacity of county and township hospitals in this region. Table 1 shows the distribution of inpatients in three MAs and medical institutions outside MAs. Ultimately, the number of inpatients in three MAs escalated from 2008 to 2015. Amongst them, the number of inpatients in MA1 increased most significantly from 10,846 in 2008 (48.42%) to 3350 in 2015 (52.79%). Although the number of inpatients in medical institutions outside MAs increased, its proportion decreased from 15.84% in 2008 to 10.26% in 2015.

**Table 1:** Distribution of inpatients in MAs from 2008 to 2015(n,%)

Year	MA1	MA2	MA3	Outside MAs	Total
2008	10,846 (48.42)	5865 (26.19)	2139 (9.55)	3548 (15.84)	22,398 (100.00)
2009	12,232 (47.46)	6851 (26.58)	2532 (9.82)	4160 (16.14)	25,775 (100.00)
2010	13,419 (50.54)	6497 (24.47)	2834 (10.67)	3803 (14.32)	26,553 (100.00)
2011	16,655 (50.91)	8356 (25.54)	3470 (10.61)	4233 (12.94)	32,714 (100.00)
2012	21,428 (48.43)	11,253 (25.43)	5071 (11.46)	6497 (14.68)	44,249 (100.00)
2013	28,249 (54.90)	11,407 (22.17)	5044 (9.80)	6751 (13.12)	51,451 (100.00)
2014	30,081 (52.98)	13,999 (24.66)	5507 (9.70)	7190 (12.66)	56,777 (100.00)
2015	33,050 (52.79)	16,092 (25.70)	7038 (11.24)	6424 (10.26)	62,604 (100.00)
Total	165,960 (51.46)	80,320 (24.90)	33,635 (10.43)	42,606 (13.21)	322,521 (100.00)

Table 2 shows the distribution of inpatients in three county hospitals. Amongst them, PH had the largest growth rate from 8461 (59.75%) in 2008 to 29,238 (67.86%) in 2015 and has been occupying the largest share of the county’s hospitalisation market. Furthermore, the number of inpatients in CMH escalated especially after 2011. However, the average proportion after the reform became lower than that before the reform. MCH slightly increased, but the proportion declined from 9.80% in 2008 to 4.95% in 2015. Since 2013, the number of inpatients in MCH evidently lessened.

**Table 2:** Distribution of inpatients in county hospitals from 2008 to 2015 (n,%)

Year	PH	CMH	MCH	Total
2008	8461 (59.75)	4312 (30.45)	1388 (9.80)	14,161 (100.00)
2009	9644 (59.76)	4989 (30.91)	1505 (9.33)	16,138 (100.00)
2010	11,991 (60.70)	5445 (27.56)	2320 (11.74)	19,756 (100.00)
2011	15,078 (61.17)	7216 (29.27)	2357 (9.56)	24,651 (100.00)
2012	18,567 (61.68)	9015 (29.95)	2522 (8.38)	30,104 (100.00)
2013	24,954 (68.37)	8920 (24.44)	2623 (7.19)	36,497 (100.00)
2014	27,365 (68.62)	10,169 (25.50)	2343 (5.88)	39,877 (100.00)
2015	29,238 (67.86)	11,716 (27.19)	2132 (4.95)	43,086 (100.00)
Total	145,298 (64.79)	61,782 (27.55)	17,190 (7.66)	224,270 (100.00)

### Three county and 10 township hospitals

Table 3 shows that the average number of inpatients per month in county and township hospitals was 1391.47 and 365.47 before the reform and 2903.68 and 708.15 after the reform, respectively. Both of the differences were statistically significant (*p* < 0.000).

**Table 3:** Independent sample t-test results

Indicators	Pre-reform	Post-reform	t-value	*P*-value
County hospitals	1391.47	2903.68	− 17.06	0.000
Township hospitals	365.47	708.15	−7.792	0.000

Table 4 shows that, the number of inpatients per month in county hospitals had an upward trend with a slope of 17.92 person/month (*P* < 0.000) before the reform and exhibited an upward trend with a slope of 31.01 person/month (P < 0.000) after the reform. The number of inpatients escalated by 265.93 at the moment of the reform (*P* = 0.002).

**Table 4:** ITSA for single-group results

Indicators	*β*_1_	SE	P-value	*β*_2_	SE	P-value	*β*_3_	SE	P-value
County	17.92	1.49	0.000	265.93	82.95	0.002	13.09	2.46	0.000
Township	−6.00	3.52	0.092	40.98	85.00	0.631	19.99	3.88	0.000

Approximately 19.99 new inpatients were admitted to township hospitals monthly after the reform (*P* < 0.000). No significant difference was observed in the instantaneous change of the reform (*P* = 0.631).

### Intervention and non-random comparison groups

Table 5 shows that before the reform, the number of inpatients in the intervention and non-random comparison groups was 236.31 and 249.14, respectively. No significant difference was observed between the two groups (*P* = 0.262). After the reform, the number of inpatients in the two groups was 521.9 and 335.23, respectively. A significant difference was observed between the two groups (*P* < 0.000).

**Table 5:** Paired t-test results

Indicators	Intervention group	Comparison group	Paired difference	t-value	*P*-value
Pre-reform	236.31	249.14	−12.83	−1.141	0.262
Post-reform	521.9	335.23	186.67	7.003	0.000

Table 6 shows the ITSA results for multiple groups between the intervention and non-random comparison groups. *β*_2_ (*P* = 0.663) and *β*_3_ (*P* = 0.469), satisfying *P* > 0.10 simultaneously, indicated no significant difference in the baseline level and baseline trend between the two groups. Therefore, complete comparability existed between them in terms of the number of inpatients.

**Table 6:** ITSA for multiple groups

Regression with Newey–West standard errors					Number of obs = 192	
Maximum lag: 2					F (7, 184) = 42.12	
					Prob > F = 0.0000	
Number of inpatients	Coef.	Std. Err.	t	P > |t|	[95% Conf. Interval]	
_t	−2.09	1.45	−1.44	0.151	−4.95	0.77
_z	24.05	55.02	0.44	0.663	−84.51	132.6
_z_t	−2.11	2.9	−0.73	0.469	−7.83	3.62
_x37	110.16	47.89	2.3	0.023	15.67	204.65
_x_t37	2.59	1.76	1.47	0.142	−0.88	6.05
_z_x37	−69.91	76.37	−0.92	0.361	− 220.59	80.77
_z_x_t37	12.56	3.26	3.85	0.000	6.13	18.99
_cons	285.75	23.82	12	0.000	238.76	332.74
Comparison of linear post-intervention trends: 37						
Linear Trend	Coeff	Std. Err.	t	P > |t|	[95% Conf. Interval]	
Treated	10.95	0.99	11.0466	0.000	8.99	12.91
Controls	0.50	0.92	0.5382	0.591	−1.32	2.32
Difference	10.45	1.35	7.7216	0.000	7.78	13.13

**Fig. 1:**
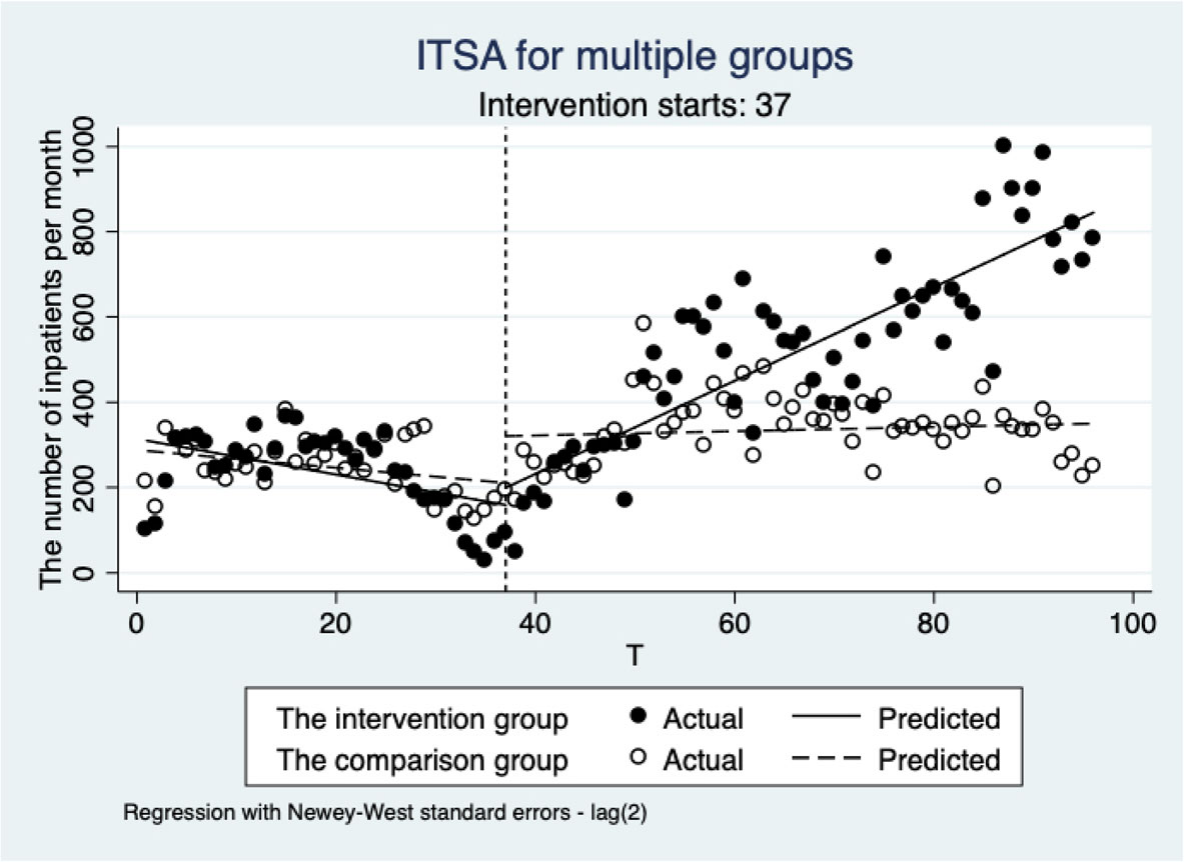
ITSA for multiple groups

After the reform, the slope of the intervention group was 10.95 person/month (*P* < 0.000), and the change of trend in the non-random comparison group showed no statistical significance (*P* = 0.5911). However, the difference between the two groups was 10.45 person/month (*P* < 0.000) indicating that the intervention group showed an increase of 10.45 new inpatients monthly compared with the non-random comparison group. Figure 1 illustrates that no significant difference occurred in the baseline level and baseline trend between the two groups before the reform. However, the growth rate of the intervention group after the reform was significantly higher than that of the non-random comparison group.

## Discussion

This reform increased the number of inpatients per month with increasing speed for three MAs. The average number of inpatients per month in three county and 10 township hospitals was 1391.47 and 365.47 before the reform and 2903.68 and 708.15 after the reform, respectively. Meanwhile, the MAs reform made medical services in three MAs more attractive to county residents [21] particularly in the utilization of health service in the township level. The township hospitals within MAs (the intervention group) substantially increased with 10.45 additional inpatients monthly compared with the township hospitals outside MAs (the non-random comparison group), which indicated that township hospitals within MAs had a more rapid development than those outside MAs.

### MAs reform increased the health service capability of township hospitals

The policy goal was to improve the weak medical service capability of primary medical institutions by establishing a resource-sharing model within the MAs. The number of inpatients in 10 township hospitals within MAs changed from negative to positive growth after the reform. In addition, the number of inpatients increased from 365.47 before the reform to 708.15 after the reform, which indicated that the reform promoted the development of primary healthcare to some extent. In the practices of MAs, the medical service capability of township hospitals improved by sinking medical resources, providing skill training for employee provided by leadership hospitals of MAs and updating medical equipment.

### Reform aggravated the unbalanced development in inpatient service amongst three MAs in the county region

The MAs reform improved the competitive advantages for each MAs [22], and the three MAs were more attractive to patients than before due to the reform, especially for MA1. We found that the number of inpatients in MA1 escalated from 10,846 (48.42%) in 2008 to 33,050 (52.79%) in 2015. The number of inpatients in PH increased from 8461(59.75%) in 2008 to 29,238 (67.86%) in 2015. However, the reform widened the gap amongst the three MAs. The continued expansion of medical market occupancy of MA1 and PH greatly decreased the living space of other MAs and medical institutions. Before the reform, the market share ratio of inpatients amongst PH, CMH and MCH was approximately 6:3:1. After the reform, the percentages of inpatients in PH, CMH and MCH changed to 67.86, 27.19 and 4.95%, respectively. Inpatient services in CMH and MCH significantly decreased and further aggravated the status of imbalance. The number of inpatients in CMH changed from 4312 (30.45%) in 2008 to 11,716 (27.19%) in 2015 and that in MCH changed from 1388 (9.80%) in 2008 to 2132 (4.95%) in 2015.

For the entire healthcare system, the original intention of MAs reform was to improve system efficiency and controlled medical expenses in the county region as a whole through medical information integration and resource sharing. However, the resource and capacity imbalance amongst PH, CMH and MCH counteracted the initial policy goal of MA reforms. The imbalance amongst the medical institutions in the county region was further aggravated [23, 24] and it implied risks of health inequality in the county’s medical market because of the concentration of high-quality medical resources. When the scale of MA1 and PH continued to expand, they would siphon off patients, doctors and other high-quality resources from the whole county. Due to the loss of high-level doctors and patients, this might cause difficulties in the development of hospitals in regions of MA2 and MA3. Ultimately, it might not only reduce the efficiency of three-level health service network, but also reduce the accessibility of residents in other areas to access basic health services, which violated the national policy of basic medical services for all.

### Shrinking business volume of CMH and MCH may cause the stagnation of medical development, thereby affecting their ability to drive subordinate township hospitals

From 2008 to 2015, the permanent population of Y county increased from 514,000 to 521,600 [25], which showed a minor increase in population. However, the compound growth rate of inpatients in Y county was 17.23%. The compound growth rate of inpatients in PH was 19.38%. And those of CMH and MCH were below the average, which were 15.35 and 6.32% respectively. The development speed of CMH and MCH was significantly slower than that of PH, and their business volume was shrinking. By 2015, the sum of business volume of CMH and MCH was less than half of that of PH.

CMH and MCH had a great competitive disadvantage with PH upon the initiation of the MAs reform. PH was a comprehensive hospital with complete departments and high-technical level, whereas CMH and MCH were specialized hospitals with medium size. Given the limited skill and medical human resources, their support and assistance to affiliated township hospitals remained restricted. Before the reform, every medical institution in the county was an independent interest body and must compete with other medical institutions to achieve their own development. However, the reform made them construct three interest communities. Each hospital in MA was not separate, and the development of the leading hospital was related to the subordinate hospitals. If the leading hospital of MAs continued to grow poorly, its support for subordinate township hospitals would be greatly limited, which might cause further delay in the development of primary healthcare institutions. Such problem was what we found through research.

### Limitations of the study

A few limitations exist in our study. Firstly, we only statistically analysed the NCMS inpatient database. Given the absence of outpatient database, we could not measure the changes in the service volume of medical institutions in a comprehensive manner. Secondly, in addition to the reform, other interfering factors between township hospitals within and outside MAs may be present, such as the inherent equipment and technology level, leaders’ ability, geographical location and traffic. These factors may have some potential effects. Thirdly, to ensure the number of interrupted points in model, we have not made periodic adjustments, which may have affected the accuracy of the statistical results. Many places in China have implemented MAs reforms, and our study only selected one sample county. Given that different regions have various environments and policy measures, our results may not be applicable to other areas where the reform has been implemented.

## Conclusion

The establishment of three MAs, where each county hospital was responsible for several township hospitals as a leading hospital, was an important attempt to integrate medical resources. The MAs reform provided valuable practical experience for medical reforms in China’s rural areas. Generally, the MAs reform has promoted the development of medical institutions in rural areas to a certain extent in county and township hospitals. After the reform, township hospitals within MAs had greater development advantages than those outside MAs in terms of the medical service capacity. However, we should still pay attention to the fact that the leading hospitals with different capabilities and levels had great differences in their ability to drive the development of township hospitals. MAs with strong leading hospitals usually took the leading position in the county. Concurrently, we should still be aware of the potential health inequality risks caused by the high concentration of high-quality medical resources and avoid the tendency of large hospitals to seize resources from primary medical institutions excessively.

## Data Availability

The datasets used and/or analysed during the current study are available from the corresponding author on reasonable request.

## Abbreviations

MAs: Medical Alliances
NCMS: New Cooperative Medical Scheme
ITSA: Interrupted Time Series Analysis
IDN: Integrated Delivery Networks
PH: People’s Hospital
CMH: Chinese Medicine Hospital
MCH: Maternal and Child Health Hospital
PCPs: Primary Care Physicians
PHOs: Physician-Hospital Organizations
MSOs: Management Services Organizations
HMOs: Health Maintenance Organizations
MA1: Medical Alliance one
MA2: Medical Alliance two
MA3: Medical Alliance three

## References

[1] Yip W, Hsiao W (2014). Harnessing the privatisation of China's fragmented health-care delivery. Lancet..

[2] Ye T, Sun X, Zhang X, Li B, Li R, Zhang L (2011). A reflection on the continuity of the three-tier healthcare network in rural China. Chin J Hosp Adm.

[3] Cheng Z, Tao H, Cai M, Lin H, Lin X, Shu Q (2015). Technical efficiency and productivity of Chinese county hospitals: an exploratory study in Henan province, China. BMJ Open.

[4] Deng F, Lv JH, Wang HL, Gao JM, Zhou ZL (2017). Expanding public health in China: an empirical analysis of healthcare inputs and outputs. Public Health.

[5] Malmivaara A (2014). On decreasing inequality in health care in a cost-effective way. BMC Health Serv Res.

[6] Cai Y, Wen C, Tang L, Liu P, Xu Y, Hu S (2018). Exploration and consideration of the medical Alliance modes. Iran J Public Health.

[7] Verhulst J, Kramer D, Swann AC, Hale-Richlen B, Beahrs J (2013). The medical alliance: from placebo response to alliance effect. J Nerv Ment Dis.

[8] Master R, Dreyfus T, Connors S, Tobias C, Zhou Z, Kronick R (1996). The community medical Alliance: an integrated system of care in greater Boston for people with severe disability and AIDS. Manag Care Q.

[9] Burns LR, Pauly MV (2002). Integrated delivery networks: a detour on the road to integrated health care?. Health Aff (Millwood).

[10] Pan J, Zhao H, Wang X, Shi X (2016). Assessing spatial access to public and private hospitals in Sichuan, China: the influence of the private sector on the healthcare geography in China. Soc Sci Med.

[11] Song H, Zuo X, Cui C, Meng K (2019). The willingness of patients to make the first visit to primary care institutions and its influencing factors in Beijing medical alliances: a comparative study of Beijing's medical resource-rich and scarce regions. BMC Health Serv Res.

[12] Zhu F, Yang Y, Wan X (2013). Practice and insight of corporate governance structure at Jiangsu Kangfu medical group. Chin J Hosp Adm.

[13] Yin H, Xie R, Ma Y, Wang C, Wang H (2017). The exploration and practice of county medical Alliance mode in Anhui Province. Chin J Health Policy.

[14] Jia Y, Fang P (2017). Effect evaluation of hierarchical diagnosis and treatment system in county -a case study of Jurong, Yicheng and Jiulongpo. Chinese Hosp.

[15] Chen Y, Li H, Gao H, Shi L, Liu L, Chang J (2017). Model and effectiveness analysis of countywide healthcare reform in Anhui province. Chin J Hosp Adm.

[16] Xia Z, Zou X, Wang Q, Liu D, Zhao L, Fang P (2014). Practices and analysis on the county-wide medical group pattern in Yicheng City. Chin J Hosp Adm.

[17] Government YMPs (2017). Yicheng national economic and social development statistical bulletin 2015 Yicheng municipal People's government.

[18] Zhang Y, Ma Q, Chen Y, Gao H (2017). Effects of public hospital reform on inpatient expenditures in rural China. Health Econ.

[19] Wagner AK, Soumerai SB, Zhang F, Ross-Degnan D (2002). Segmented regression analysis of interrupted time series studies in medication use research. J Clin Pharm Ther.

[20] Linden A (2018). Conducting interrupted time-series analysis for single- and multiple-group comparisons. Stata J.

[21] Sperl-Hillen JM, O'Connor PJ (2005). Factors driving diabetes care improvement in a large medical group: ten years of progress. Am J Manag Care.

[22] Sowa PM, Kault S, Byrnes J, Ng SK, Comans T, Scuffham PA (2018). Private health insurance incentives in Australia: in search of cost-effective adjustments. Appl Health Econ Health Policy.

[23] Casalino LP (2006). Which type of medical group provides higher-quality care?. Ann Intern Med.

[24] Kralewski JE, Zink T, Boyle R (2012). Factors influencing electronic clinical information exchange in small medical group practices. J Rural Health.

[25] Government YMPs (2009). Yicheng national economic and social development statistical bulletin 2008 Yicheng municipal People’s government.

